# Effectiveness of Nusinersen in Type 1, 2 and 3 Spinal Muscular Atrophy: Croatian Real-World Data

**DOI:** 10.3390/jcm12082839

**Published:** 2023-04-13

**Authors:** Andrej Belančić, Tea Strbad, Marta Kučan Štiglić, Dinko Vitezić

**Affiliations:** 1 Department of Clinical Pharmacology, Clinical Hospital Centre Rijeka, 51000 Rijeka, Croatia; dinko.vitezic@uniri.hr; 2 Department of Basic and Clinical Pharmacology with Toxicology, Faculty of Medicine, University of Rijeka, 51000 Rijeka, Croatia; 3 Croatian Health Insurance Fund, 10000 Zagreb, Croatia; tea.strbad@hzzo.hr; 4 Primorje-Gorski Kotar County Community Health Centre, 51000 Rijeka, Croatia; marta.kucan@ri.t-com.hr

**Keywords:** effectiveness, motor function, nusinersen, real-world data, spinal muscular atrophy

## Abstract

(1) Background: To investigate the real-world effectiveness and safety profile of nusinersen in Croatian paediatric and adult spinal muscular atrophy (SMA) patients. (2) Methods: A retrospective and anonymous collection of relevant demographic and clinical data for all Croatian SMA patients treated with nusinersen and reimbursed by the Croatian Health Insurance Fund (CHIF) between April 2018 and February 2022 was performed through searching the CHIF database and studying the associated reimbursement documentation. All patients who received at least one dose of nusinersen were included in the baseline clinical-demographic overview and safety analysis, whereas only subjects who had completed six doses were included in the effectiveness analysis. (3) Results: Fifty-two patients [61.5% male; median age 13.4 (0.1–51.1) yr.] received nusinersen treatment. In SMA type 1 and type 3 paediatric patients, statistically significant motor function improvement (CHOP INTEND 10.8 ± 10.3 vs. 20.0 ± 15.8, *p* = 0.003; HFMSE 49.6 ± 7.9 vs. 53.1 ± 7.7, *p* = 0.008; respectively) was achieved immediately after 4 loading doses of nusinersen and remained statistically significant onwards. Average improvements in HFMSE motor performance in SMA type 2 patients after four, five, and six doses of nusinersen were +6.0, +10.5, and +11.0 points, respectively. In SMA type 3 adult patients, no significant improvement in RHS motor performance or the 6-Minute Walk Test (MWT) was demonstrated. During the study period, 437 doses were administered without any new safety concerns appearing. (4) Conclusions: Our RWD findings indicate that nusinersen is an effective and safe treatment in a heterogeneous group of paediatric patients with all types of SMA; however, no significant benefit (but only RHS and 6MWT maintenance) was demonstrated in SMA type 3 patients who started nusinersen after >18 years of age.

## 1. Introduction

Spinal muscular atrophy (SMA) is a rare hereditary motor neuron disorder, with an estimated prevalence of 1 or 2 in every 100,000 persons and an incidence of approximately 1 in every 10,000 live births, caused by an insufficient level of survival motor neuron (SMN) protein due to SMN1 gene homozygous deletion or mutation [1,2]. The SMN2 gene is a disease modifier that has a partial function and is able to compensate for homozygous deletions of SMN1 to some extent. Copy number variants of exon 7 of SMN2 are also meaningful in the prediction of patients’ responses to disease-modifying drugs (DMDs) [3,4]. The copy number of the neuronal apoptosis inhibitory protein (NAIP) gene, which is located on chromosome 5q13.2, has also been linked to the severity of SMA (although alone it is not a sufficient predictor), since concurrent homozygous SMN1 deletion and NAIP deletion are linked with early onset, severe hypotonia, and a worse outcome or course of the disease [3,4,5]. 

SMA is characterised by degeneration of motor neurons in the spinal cord, leading to progressive muscle weakness and even respiratory insufficiency in the most severe cases (a heterogeneous clinical phenotype) [1]. Based on age at symptom onset, maximum motor milestone acquired, and severity of symptoms, it can be classified into five types (0–4), with SMA type 1 accounting for nearly 60% of cases (Table 1) [2,6,7]. 

SMA patients require multidisciplinary care due to the need to cover all aspects of the diagnosis and its management, primarily focused on neurological, neuro-muscular rehabilitation, orthopaedic, pulmonary, and nutritional care [8,9]. Nusinersen (Spinraza^®^), an antisense oligonucleotide for intrathecal administration that increases the proportion of exon 7 inclusion in SMN2 (which is a disease modifier) mRNA transcripts and ultimately promotes the production of full-length SMN protein, was the first DMD approved for the treatment of 5q SMA [6,10,11]. The efficacy of nusinersen, regarding motor response and event-free survival in infantile-onset and later-onset SMA patients, has been proven in two phase III clinical trials (ENDEAR and CHERISH) [12,13]. Nusinersen was approved by the Food and Drug Administration (FDA) in December 2016, while the European Medicines Agency (EMA) did it in June 2017. Although registered for all types of 5q SMA, data regarding the nusinersen therapeutic effect in SMA type 3 patients is still relatively scarce and predominantly based on real-world data (RWD) findings [10,14].

Randomised clinical trials (RCTs) in rare diseases generally include small, homogeneous groups of patients. This is the reason why various patient populations are under-represented in clinical trials and RWD collection and disease registry networks are essential to support orphan medication’s effectiveness, safety, and tolerability [15]. The objective of our research was to investigate the real-world effectiveness and safety profile of nusinersen in Croatian paediatric and adult SMA patients.

## 2. Materials and Methods

A retrospective and anonymous collection of relevant demographic and clinical (effectiveness and safety) data for all Croatian SMA patients treated with nusinersen and reimbursed by the Croatian Health Insurance Fund (CHIF) between April 2018 and February 2022 was performed through searching the CHIF database and studying the associated reimbursement documentation. Based on the initial (24 April 2018) CHIF nusinersen administration criteria, treatment costs could have been reimbursed for SMA types 1–3 patients who were: (i) <18 yr. old; (ii) not on mechanical ventilation (MV); and (iii) have ≥2 SMN2 copy numbers. In addition, reimbursement guidelines and criteria changed, and since 12 July 2019, the cost of nusinersen treatment can be reimbursed for all SMA patients, irrespective of age, need for MV, or number of SMN2 copy numbers. For reimbursement purposes, a request from the Drug and Therapeutics Committee of the National Referral Centre for SMA, accompanied by relevant clinical documentation, has to be submitted to CHIF, and this includes an initial request for four loading doses and then an individual request every four months for each maintenance dose. Data regarding age when nusinersen was introduced, gender, SMA type and stage, SMN1, SMN2, and NAIP copy numbers, number of nusinersen doses received and its associated overall cost during the study period, baseline motor function, motor function response, need for respiratory (MV or bi-level positive airway pressure—BiPAP) and feeding (percutaneous endoscopic gastrostomy—PEG or nasogastric tube) support initially and throughout the study period, as well as safety data, were collected for each subject under a de-identified personal code in a patient-specific form, from the documentation. Through the CHIF database, we had access to the baseline assessments (just before the administration of the first nusinersen dose) and to the assessments prior to the administration of each following injection—starting from the 5th dose administration (day 185 of the treatment). When it comes to the motor function evaluations, CHOP INTEND (Children’s Hospital of Philadelphia Infant Test of Neuromuscular Disorders) was available for all non-sitting patients and those younger than 2 yr. of age. HFMSE (Hammersmith Functional Motor Scales Expanded)-based evaluations were available for all sitters, as well as walkers who were < 18 yr. old (SMA 3p), while adult walkers (SMA 3a) had motor function evaluations based on the RHS (Revised Hammersmith Scale) protocol, which was for some patients accompanied by the 6-Minute Walk Test. Data of all patients who received at least one dose of nusinersen (reimbursed by CHIF) were included in the baseline clinical-demographic overview and safety analysis, whereas only subjects who had completed the first six nusinersen doses without missing follow-up data were included in the effectiveness (motor, respiratory, and feeding) analysis. For SMA 3p patients who had at some point transitioned from paediatric to adult neurologic care (SMA 3pa), motor effectiveness analyses were performed up to the point when the last assessment by HFMSE was available.

Collected data were imported into Microsoft Excel 2016 (Microsoft Office) and MedCalc v12.1.3 (MedCalc Software bvba, Ostend, Belgium), which were subsequently used for statistical analysis. The Kolmogorov-Smirnov test was used to assess the normality of the distribution. Variable values were described by absolute and relative frequencies, measures of central tendency, and measures of spread. Changes over time in terms of motor function were analysed with the Wilcoxon matched pairs signed rank test for each SMA type separately. The associations between the change from baseline in motor function (evaluation after 5 doses of nusinersen already received) and the age of treatment initiation were assessed by Spearman’s correlation. Overall, the criterion for statistical significance was set at *p* < 0.05.

The study was conducted in accordance with the Declaration of Helsinki. Bearing in mind the retrospective nature of the study and its design, as well as the fact that the used data had already been anonymized and recorded, a waiver of informed consent was approved.

## 3. Results

During the study period, 52 patients [61.5% male; median age 13.4 (0.1–51.1) yr.] received nusinersen treatment reimbursed by CHIF. The patients’ baseline demographic and clinical characteristics are presented in Table 2. Baseline data showed that twelve patients required MV (10 SMA type 1 patients and two SMA type 2 patients), and eight children (all SMA type 1) required BiPAP support. According to baseline feeding device support, PEG was required by four SMA type 1 patients, while a nasogastric tube was needed in eight SMA type 1 and two SMA type 2 patients (Table 2).

**Figure 1:**
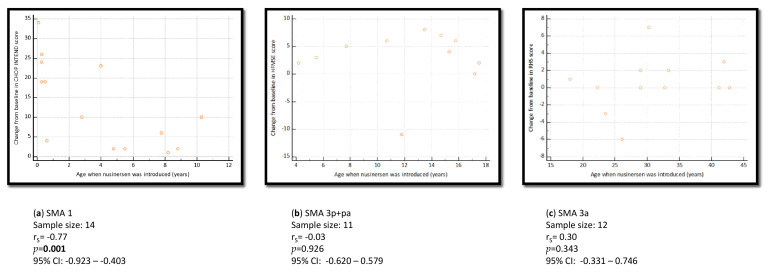
(**a**–**c**): The association between the change from baseline in motor function (after 5 doses of nusinersen) and the age at treatment initiation. (**a**) SMA type 1 patients; (**b**) SMA type 3p+pa patients; (**c**) SMA type 3a patients.

Motor function improved in all 14 SMA type 1 patients that were included in the motor effectiveness analysis after five doses of nusinersen were received, i.e., 33.3% (2/6) of SMA type 1 patients on MV support and 100% (8/8) of those who were not on MV improved their CHOP INTEND motor function by ≥4 points. The change from baseline became statistically significant at the time of the evaluation just before the 5th injection and remained statistically significant onward (Table 3). There was a negative correlation (*p* = 0.001, rS = −0.77, 95% CI: −0.923–−0.403) between the age at treatment initiation and the change from baseline in motor function, as presented in Figure 1a. According to subanalysis findings, nusinersen’s effectiveness, regarding motor function improvement, was demonstrated both for SMA type 1 patients on MV and those without (Table 3). Precisely, six SMA type 1 patients on MV were eligible for the latter effectiveness subanalysis (initial CHOP INTEND 3.8 ± 3.4); their average improvements in motor performance after four, five, and six doses of nusinersen were +1.2 (5.0 ± 2.9; *p* = 0.375), +3.9 (7.7 ± 4.2; *p* = 0.031), and +4.9 points (8.7 ± 4.6; *p* = 0.031), respectively.

Only 2/6 SMA type 2 patients were eligible for the effectiveness analysis (initial HFMSE 8.5 ± 12.0). Thus, due to the small sample size, the Wilcoxon matched pairs signed rank test as well as Spearman’s correlation analyses could not have been performed. However, it is worth mentioning that the average improvements in motor performance (measured by HFMSE) after four, five, and six doses of nusinersen were +6.0 (14.5 ± 17.7), +10.5 (19.0 ± 24.0), and +11.0 points (19.5 ± 24.7), respectively.

All 11 SMA type 3p+pa patients were included in the motor performance improvement analysis. By the time of the 6th injection, motor function (HFMSE) had improved in 81.8% (9/11) of patients, and the improvement was ≥4 points in 66.7% (6/9) of such cases. The change from baseline became statistically significant immediately after 4 loading doses of nusinersen were received, and it remained statistically significant during the study period (Table 4). There was no association between the age at treatment initiation and the change from baseline in motor function (*p* = 0.926, rS = −0.03, 95% CI: −0.620–0.579) (Figure 1b).

When it comes to SMA type 3a patients, 12/17 met the criteria to be included in the motor effectiveness analysis. As presented in Table 5, statistically significant improvement in motor performance (measured by RHS) was not achieved with nusinersen treatment. There was no significant improvement in terms of the 6-Minute Walk Test [N = 4 patients; before treatment: 397.5 (304–413) m vs. after 8 doses already received: 411.0 (380–519) m; *p* = 0.250]. What is more, there was no association between the age at treatment initiation and the change from baseline in motor function (*p* = 0.343, rS = 0.30, 95% CI: −0.331–0.746) (Figure 1c). 

Respiratory function worsening occurred only in one SMA type 2 patient in whom BiPAP was introduced after five doses of nusinersen. Feeding worsening occurred in one SMA type 1 patient (who initially had a nasogastric tube) in whom PEG was introduced after 13 doses received. There was one non-drug-related lethal event during the study period. It was a SMA type 1 patient (who was on BiPAP and needed a nasogastric tube for feeding) who died at 3.9 yr. of age and in whom CHOP INTEND improved from 12 to 44 after 13 doses of nusinersen were received.

Overall, 437 injections of nusinersen were administered. The adverse drug reactions (ADRs) profile in our study showed the same safety and tolerability profile for nusinersen as demonstrated in clinical trials; no new safety concerns have emerged. Headache, back pain, and vomiting were the only ADRs identified; their frequencies (≥1/10 patients) were similar to those presented in the summary of product characteristics. During the study period, 57.7% (N = 30) of patients were switched to risdiplam at some point.

## 4. Discussion

Our RWD findings indicate that nusinersen is an effective treatment in a heterogeneous group of paediatric patients with all types of SMA; however, no significant benefit (but only RHS and 6MWT maintenance) was demonstrated in SMA type 3 patients who started nusinersen after more than 18 years of age. 

All SMA type 1 patients improved their motor function after five doses of nusinersen were received, which is in accordance with RWD from Hungarian patients recently published by Szabó et al. At that time point, the average improvement in CHOP INTEND was 13.0 for our cohort versus 14.9 points for the Hungarian cohort [14]. Moreover, other RWD reports also speak in favour of nusinersen’s effectiveness in SMA type 1 patients [16,17,18]. The advantage of our study is a separate subanalysis for SMA type 1 patients on and not on MV, which demonstrated nusinersen’s effectiveness in both groups. Expectedly, slighter improvements were observed for those requiring MV support (+3.9 points after five doses of nusinersen). 

SMA type 1 patients who started nusinersen treatment earlier achieved better outcomes in terms of motor response (Figure 1a). Similar findings were reported by Pane et al. and Pechmann et al., who observed more obvious motor function improvements in SMA patients aged less than or equal to 7 months compared to older children [19,20]. This is not surprising since untreated infantile-onset SMA is characterised by early loss of motor function and compound muscle action potentials as well as early complication development. This is the result of the rapid progression of the degeneration of motor neurons in the spinal cord. Thus, implementing a test for 5q SMA for all new-born children into a Croatian national screening programme might allow us to start DMD treatment earlier (on time), preventing motor neuron degeneration, the development of severe disabilities, complications, or even death in the latter subpopulation [21,22].

The efficacy of nusinersen in SMA type 2 patients was first demonstrated in the CHERISH randomised controlled trial. Mercuri et al. reported improvement of HFMSE by at least three points after 15 months of follow-up in 57% of children with later-onset SMA treated with nusinersen (versus 26% in the control group) [13]. The interim report of the open-label extension of the latter study (SHINE) also points towards long-term efficacy, while a final analysis report is still expected [23]. Our RWD findings are in favour of nusinersen’s effectiveness in SMA type 2 patients (average improvements in HFMSE after four, five, and six doses of +6.0, +10.5, and +11.0, respectively); however, we could not perform the Wilcoxon matched pairs signed rank test due to the low sample size. Although our results point towards a positive trend, they do not provide a final statistical conclusion for our SMA type 2 cohort. In addition, Hungarian RWD reported by Szabó demonstrated +4.0, +7.2, and +7.0 points, a significant improvement of HFMSE after four to six doses of nusinersen, respectively. A negative correlation between the age at treatment initiation and treatment outcomes was also highlighted in the study [14]. Pane et al. recently published an Italian RWD study and showed increases in motor function between baseline and 12-month as well as 24-month periods, with more obvious changes in younger patients with higher baseline motor function [24]. Furthermore, RWD from a Slovenia-Czech Republic combined research group as well as authors from Switzerland all concluded the effectiveness of nusinersen in SMA type 2 [16,18]. Lastly, nusinersen’s favourable net benefit in motor function across a range of SMA type 2 patients was also presented in a recent critical review and meta-analysis published by Coratti et al. [25]. 

We provided separate analyses for SMA type 3 paediatric and adult patients, since their motor function was clinically assessed with different motor scales (also available in the CHIF database)—the HFMSE and the RHS, respectively. When it comes to our SMA type 3 paediatric patients, the change from baseline in HFMSE became statistically significant immediately after four loading doses (+3.5 points) and remained statistically significant onward (Table 4). By the time of the sixth injection, almost 82% of patients had improved their motor function compared to the baseline. Similarly, Szabó et al. found an average HFMSE improvement of 5.3 points by the time of the sixth injection and concluded that the drug had significant real-world effectiveness [14]. On the contrary, in a study by Osredkar et al., only a trend towards improvement was shown after 14 months of nusinersen treatment, while in a study by Pane et al., significant improvement in HFMSE was not reached after 12 months but after 24 months (+1.5 point; *p* = 0.017) of nusinersen treatment [16,24]. 

On the other hand, no significant improvement in RHS (+0.5 by the time of the 6th injection) or the 6-Minute Walk Test (+13.5 m) was achieved in a cohort of SMA type 3 patients in whom nusinersen was introduced at >18 years of age. To compare, in a prospective study by Walter et al., only a mild nusinersen effect (6-Minute Walk Test after days 180 and 300 and RULM after day 300) was demonstrated in adults (18–59 years of age) with long-standing SMA type 3 after 10 months of treatment, and there were no notable improvements in motor function measured by the HFMSE [26]. Coratti et al. pointed towards a favourable benefit of nusinersen in a wide range of SMA type 2 and 3 patients; however, it is worth mentioning that their sample was very heterogeneous, combining both paediatric and adult patients, ambulant and non-ambulant patients, as well as both SMA types [25]. Thus, we believe that more well-designed studies are still highly needed to determine the absolute result regarding nusinersen’s effectiveness in SMA type 3 when introduced after 18 years of age.

RCTs in rare diseases do not represent the full clinical spectrum of patients in the real world. Hence, real-world evidence plays an increasing role in regulatory decisions, especially for medicines such as those for very rare diseases and “precision medicines” [27]. So, RWD are supportive of an orphan drug’s safety and efficacy on a wider, heterogeneous sample and want to gain further insights and experience with DMTs’ individualization. This then results in the desired modification of the disease course (improvement and/or perseverance of functions) and maximisation of patients’ quality of life, participation, and independence. On top of that, patient registries and RWD can also be used to better understand disease natural histories, improve standards of care, provide opportunities to connect patients with the research community, and monitor patient outcomes, both from a clinical and regulatory perspective. Outcome and cost-effectiveness data is definitely highly valuable for national health insurance providers who can compose and regularly modify their therapy reimbursement guidelines, as well as optimise and adequately distribute financial resources based on such findings.

To deduce, 25.40% (111/437) of the nusinersen doses administered during the study period were for SMA type 3 adult patients, which corresponds to a total cost of approximately 501,385.58 euros. Thus, long-term prospective studies are highly desirable to draw final conclusions regarding the nusinersen effect as well as cost-effectiveness for this SMA subpopulation. Subsequent future (re)evaluation might result in modification of the national health insurance fund’s nusinersen and DMD administration criteria and reimbursement guidelines and a shift of the accompanied financial resources to other indications where more substantial clinical benefit was proven.

Lastly, it is worth mentioning that almost 60% of patients were switched from nusinersen to risdiplam at some point in time. Further follow-up of such patients might eventually give us valuable insights and experience with the DMD switch in terms of relative effectiveness (motor and regarding patient-reported outcomes), safety, and treatment personalization, taking into consideration multiple individual patients’ characteristics.

This was a retrospective, observational study using aggregated data from the CHIF database and reimbursement documentation; thus, the findings should be interpreted accordingly. The limitation lays in the fact that there is a chance that some AEs that happened during the treatment were potentially not mentioned in the medical records and that the disease course (motor function answer) was not prospectively followed by the same assessor for all patients included. However, all patients were strictly followed in the same medical centre (Clinical Hospital Centre Zagreb), and motor function was at all times estimated by the starting or initial well-trained assessor using the appropriate and standard motor scale in order to obtain uniformity. Moreover, it would be valuable to obtain findings regarding patient-reported outcome measures in order to get better insights on the impact on patients’ quality of everyday lives; however, this was not possible because of the study design and the unavailability of such measures. The strengths of this study lie in the fact that the presented results are based on data from all Croatian paediatric and adult SMA patients treated with nusinersen and reimbursed by the CHIF over a four-year follow-up period. On top of that, detailed patients’ genotype data, individual motor effectiveness subanalyses for SMA type 1 patients regarding MV requirement, and individual analyses for both SMA type 3p and type 3a patients are reported here. This is a notable advantage since it was not a standard case within the previous body of literature generally.

## 5. Conclusions

According to our real-world data, nusinersen seems to significantly improve motor function in SMA type 1 (irrespective of MV support), SMA type 2, and SMA type 3p patients. On the contrary, no significant improvement (but only maintenance) in RHS or the 6-Minute Walk Test was achieved in SMA type 3a patients; thus, more long-term prospective studies are still required to draw final conclusions regarding nusinersen’s cost-effectiveness in the SMA type 3a subpopulation. What is more, nusinersen demonstrated a similar safety and tolerability profile as noted in clinical trials and the summary of product characteristics documentation. Last but not least, there is still an unmet need for continuous regional and local data collection as well as disease registry networks to gain further insights into the natural histories of individual SMA types and the (cost)effectiveness and safety of nusinersen and DMTs in the patient subpopulations under-represented in RCTs, especially now that even newer and more expensive therapeutic options for SMA are available on the market.

## Figures and Tables

**Table 1:** Clinical classification of SMA based on age at symptom onset, maximum motor milestone acquired, severity of symptoms, and clinical continuum (before DMDs).

SMA Type	Age at Symptom Onset	Maximum Motor Function Achieved	Prognosis
0	Prenatal/foetal	Nil	Poor; usually die before 6 months
1	<6 months	Non-sitter	Poor; usually die before 2 years
2	7–18 months	Sit independently	Expected to live into their twenties and beyond
3	>18 months	Walker	Progressive weakness, motor disability, normal lifespan
4	10–30 yr	Walker	Weakness of lower extremities, normal lifespan

**Table 2:** Overview of baseline demographic and clinical parameters of all patients, including subgroups according to SMA types.

Baseline Demographics		SMA 1		SMA 2		SMA 3p+pa		SMA 3a		SMA 3 Total	Total
**N (% out of total)**		18 (34.6%)		6 (11.5%)		11 (21.1%)		17 (32.7%)		28 (53.8%)	52 (100%)
**Age** (years)	X ± SD	6.3 ± 6.3		8.8 ± 6.0		12.2 ± 4.6		32.4 ± 8.5		24.5 ± 12.3	16.4 ± 13.2
	Med (Min-Max)	5.2 (0.1–20.8)		10.2 (1.5–16.7)		13.5 (4.2–17.5)		30.5 (18–51.1)		24.8 (4.2–51.1)	13.4 (0.1–51.1)
**Male/female**		12/6		3/3		6/5		11/6		17/11	32/20
**SMN1 exon 7 copy number**	0	17		5		11		17		28	50
	1	1		1		0		0		0	2
**SMN1 exon 8 copy number**	0	14		4		7		12		19	37
	1	4		2		4		2		6	12
	2	0		0		0		3		3	3
**SMN2 exon 7 copy number**	1	1		1		0		0		0	2
	2	15		1		2		0		2	18
	3	2		4		6		4		10	16
	4	0		0		3		13		16	16
**SMN2 exon 8 copy number**	1	4		1		0		0		0	5
	2	12		1		1		4		5	18
	3	2		4		6		4		10	16
	4	0		0		4		9		13	13
**NAIP copy number**	0	9		0		1		0		1	10
	1	8		6		2		3		5	19
	2	1		0		8		13		21	22
	3	0		0		0		1		1	1
**Mechanical ventilation**—N (% out of total for corresponding SMA type)		10 (55.6%)		2 (33.3%)		0 (0%)		0 (0%)		0 (0%)	12 (23.1%)
**BiPAP**—N (% out of total for corresponding SMA type)		8 (44.4%)		0 (0%)		0 (0%)		0 (0%)		0 (0%)	8 (15.4%)
**PEG**—N (% out of total for corresponding SMA type)		4 (22.2%)		0 (0%)		0 (0%)		0 (0%)		0 (0%)	4 (7.7%)
**NG tube**—N (% out of total for corresponding SMA type)		8 (44.4%)		2 (33.3%)		0 (0%)		0 (0%)		0 (0%)	10 (19.2%)
**Baseline motor function** (N of patients)	X ± SD	CHOP INTEND (N = 17)	9.1 ± 10.1	HFMSE (N = 4)	5.8 ± 8.0	HFMSE (N = 11)	49.6 ± 7.9	RHS (N = 15)	48.5 ± 15.3	N/A, N = 26	N/A, N = 47
	Med (Min-Max)		5 (0–38)		3 (0–17)		51 (29–60)		52 (19–65)		

SMA—spinal muscular atrophy; a—adult; p—paediatric; pa—paediatric patients that were at some point transitioned from paediatric to adult neurologic care; MV—mechanical ventilation; BiPAP—bi-level positive airway pressure; PEG—percutaneous endoscopic gastrostomy; NG—nasogastric; CHOP INTEND—Children’s Hospital of Philadelphia, Infant Test of Neuromuscular Disorders; HFMSE—Hammersmith Functional Motor Scales Expanded; RHS—Revised Hammersmith Scale. To the best of our knowledge, there are 16 SMA 2 patients in Croatia; however, according to our database, nusinersen was ultimately reimbursed by Croatian Health Insurance Fund for 6 patients that are presented in Table 2.

**Table 3:** The average CHOP INTEND scores by the time points of the nusinersen injection in SMA type 1 patients.

Time of Evaluation/Doses Already Received	All SMA Type 1 Patients				SMA Type 1 Patients Not on MV			
	N of Patients	CHOP INTEND/X ± SD	Change from Baseline	*p* Value	N of Patients	CHOP INTEND/X ± SD	Change from Baseline	*p* Value
Before treatment	14	10.8 ± 10.3	N/A	N/A	8	16.0 ± 10.8	N/A	N/A
Day 185/4	14	20.0 ± 15.8	9.2	**0.003**	8	31.3 ± 11.0	15.3	**0.016**
Day 307/5	14	23.8 ± 17.0	13.0	**<0.001**	8	35.9 ± 11.6	19.9	**0.008**
Day 429/6	14	25.4 ± 18.6	14.6	**<0.001**	8	37.9 ± 14.5	21.9	**0.008**
Day 551/7	11	29.6 ± 18.2	18.2	**0.001**	7	40.9 ± 12.0	25.7	**0.016**
Day 673/8	7	36.0 ± 16.3	22.5	**0.016**	6	40.5 ± 12.2	24.8	**0.031**
Day 795/9	7	36.4 ± 18.6	22.9	**0.016**	6	41.0 ± 15.5	25.3	**0.031**
Day 917/10	7	40.1 ± 18.6	26.6	**0.016**	6	45.3 ± 13.8	29.6	**0.031**
Day 1039/11	5	46.0 ± 15.1	31.6	0.062	5	46.0 ± 15.1	31.6	0.062
Day 1161/12	4	44.3 ± 13.5	35.8	0.125	4	44.3 ± 13.5	35.8	0.125
Day 1283/13	3	44.7 ± 15.9	37.4	N/A	3	44.7 ± 15.9	37.4	N/A
Day 1405/14	3	44.0 ± 15.1	36.7	N/A	3	44.0 ± 15.1	36.7	N/A
Day 1527/15	3	45.3 ± 13.9	38.0	N/A	3	45.3 ± 13.9	38.0	N/A

**Table 4:** The average HFMSE scores by the time points of the nusinersen injections in paediatric SMA type 3 patients.

Time of Evaluation/Doses Already Received	N of Patients	HFMSE/X ± SD	Change from Baseline	*p* Value
Before treatment	11	49.6 ± 7.9	N/A	N/A
Day 185/4	11	53.1 ± 7.7	3.5	**0.008**
Day 307/5	11	52.5 ± 9.7	2.9	0.084
Day 429/6	11	54.7 ± 9.0	5.1	**0.049**
Day 551/7	9	55.1 ± 9.2	6.1	**0.008**
Day 673/8	9	55.2 ± 9.5	6.2	**0.020**
Day 795/9	8	55.1 ± 7.9	6.5	**0.023**
Day 917/10	8	55.4 ± 8.2	6.8	**0.016**
Day 1039/11	6	55.8 ± 8.2	8.1	**0.031**
Day 1161/12	6	56.2 ± 8.3	8.5	**0.031**
Day 1283/13	2	59.0 ± 4.2	8.0	N/A

**Table 5:** The average RHS scores by the time points of the nusinersen injections in adult SMA type 3 patients.

Time of Evaluation/Doses Already Received	N of Patients	RHS/X ± SD	Change from Baseline	*p* Value
Before treatment	12	47.9 ± 16.1	N/A	N/A
Day 185/4	12	47.2 ± 15.8	−0.7	0.313
Day 307/5	12	48.4 ± 15.9	0.5	0.578
Day 429/6	12	48.5 ± 16.2	0.6	0.577
Day 551/7	10	51.4 ± 17.1	1.5	0.250
Day 673/8	9	51.7 ± 18.5	2.0	0.055

## Data Availability

The datasets generated during and/or analysed during the current study are available from the corresponding author on reasonable request.
